# Enhanced angiogenic function in response to fibroblasts from psoriatic arthritis synovium compared to rheumatoid arthritis

**DOI:** 10.1186/s13075-019-2088-3

**Published:** 2019-12-21

**Authors:** S. Fromm, C. C. Cunningham, M. R. Dunne, D. J. Veale, U. Fearon, S. M. Wade

**Affiliations:** 1 Department of Molecular Rheumatology, Trinity Biomedical Sciences Institute, Trinity College Dublin, Dublin, Ireland; 2 Department of Surgery, Trinity Translational Medicine Institute, St. James’s Hospital, Trinity College Dublin, Dublin, Ireland; 3 Rheumatology EULAR Centre of Excellence, Centre for Arthritis & Rheumatic Diseases, University College Dublin, Dublin, Ireland

**Keywords:** Angiogenesis, Fibroblasts, Psoriatic arthritis, Rheumatoid arthritis

## Abstract

**Introduction:**

Angiogenesis is an early event in the pathogenesis of both psoriatic arthritis (PsA) and rheumatoid arthritis (RA); however, there are striking differences in blood vessel morphology and activation between the two arthropathies. The aim of this study was to assess if the PsA and RA joint microenvironments differentially regulate endothelial cell function.

**Methods:**

PsA and RA primary synovial fibroblasts (SFC) were isolated from synovial biopsies, grown to confluence, and supernatants harvested and termed ‘conditioned media’ (CM). Human umbilical vein endothelial cells (HUVEC) were cultured with PsA SFC or RA SFC-CM (20%). HUVEC tube formation, migration, and PBMC adhesion were assessed by matrigel tube formation, wound repair, and PBMC adhesion assays. HUVEC cell surface expression of ICAM, VCAM, and E-Selectin was assessed by flow cytometry. Transcriptome analysis of genes promoting angiogenesis was performed by real-time PCR. Finally, a MSD multiplex angiogenic assay was performed on PsA SFC and RA SFC supernatants.

**Results:**

Macroscopic synovitis and vascularity were similar in PsA and RA patients; however, significant differences in vascular morphological pattern were recorded with tortuous, elongated vessels observed in PsA compared to straight regular branching vessels observed in RA. Transcriptome analysis showed strong upregulation of the pro-angiogenic signature in HUVEC primed with PsA SFC-CM compared to RA SFC-CM and basal control. In parallel, paired PsA SFC-CM significantly induced HUVEC tube formation compared to that of RA SFC-CM. Furthermore, PsA SFC-CM induced HUVEC migration was paralleled by a significant induction in VEGFA, PFKFB3, ICAM-1, and MMP3 mRNA expression. A significant increase in PBMC adhesion and cell surface expression of VCAM-1, ICAM-1, and E-Selectin expression was also demonstrated in PsA SFC-CM-primed HUVEC compared to RA SFC-CM. Finally, VEGF, TSLP, Flt-1, and Tie-2 expression was elevated in PsA SFC-CM compared to RA SFC-CM, with no significant difference in other pro-angiogenic mediators including MIP-3, bFGF, PIGF, and MCP-1.

**Conclusion:**

PsA SFC and RA SFC secreted factors differentially regulate endothelial cell function, with soluble mediators in the PsA joint microenvironment inducing a more pro-angiogenic phenotype compared to the RA.

## Introduction

Dysregulated angiogenesis is an early event in inflammatory arthritis (IA), facilitating leukocyte recruitment and synovial membrane hyperplasia and creating an aggressive pannus tissue capable of destroying adjacent cartilage and bone [1]. We and others have previously demonstrated distinct macroscopic vascular morphology in the joints of inflammatory arthritis. PsA synovial vasculature is characterised as elongated, tortuous vessels with minimal branching, while RA synovia display straight, regular branching vessels [1–4]. While the observed tortuous vascular pattern in PsA is a consistent finding, studies have shown in RA that rheumatoid factor (RF) positivity is associated with the regular straight vascular pattern, while RA RF negative patients can display a tortuous pattern [2–4]. At a microscopic level, an increase in blood vessel number has also been demonstrated by many studies [4–6], which may be due to the elongation and increased tortuosity of existing vessels, rather than an increase in the actual number of new vessels. These changes are associated with differential circulatory and synovial expression of angiogenic factors, such as vascular endothelial growth factor (VEGF), angiopoietin-2 (Ang2), placental growth factors (PIGF), and stromal derived growth factor-1 (SDF-1), in addition to cytokines and matrix metalloproteinases (MMPs) [1, 6–10]. Increased expression of these growth factors has been demonstrated in early PsA synovial membrane compared to RA [7, 9, 11], suggesting that mechanisms involved in regulating the distinct vascular morphology in PsA occur at an early stage of disease.

The synovial microenvironment, which is composed of stromal cells, immune cells, endothelial cells, and extracellular matrix components, plays an important role in disease progression in inflammatory arthritis. Synovial fibroblast cells (SFC) are a crucial cell population in the inflamed synovium microenvironment and promote the initiation and progression of joint destruction, immune cell invasion, and angiogenesis. SFC interact with the cells within the inflamed synovium through the secretion of multiple pro-inflammatory factors that regulate their pathologic activities [12]. In conjunction with this, fibroblasts are also thought to play a key role in angiogenesis. Numerous studies have shown that SFC secrete multiple soluble angiogenic growth factors including VEGF, angiopoietins, basic fibroblast growth factor (bFGF), and MMP2/9 [12–18]. Moreover, vascularisation of matrigel implants in immune deficient mice is increased in those containing RA SFC compared to both HC and OA SFC [19]. While these studies demonstrate that SFC play a role in regulating angiogenic mechanisms, the differing contribution of RA and PsA SFC to the process of joint angiogenesis remains largely unknown. In the present study, we aimed to identify if RA and PsA SFC differ in their capacity to induce pro-angiogenic activity of endothelial cells. Using SFC-CM derived from RA and PsA patients with matched macroscopic scores of synovial inflammation (synovitis) and angiogenesis, we demonstrate that culture of endothelial cells with PsA SFC-CM enhanced migration, tube formation, surface expression of adhesion markers, and immune cell adhesion, to a significantly greater capacity than culturing with RA SFC-CM. These data suggest that PsA SFC may contribute, in part, to the distinct vascular pattern observed in the PsA synovium.

## Methods

### Patient recruitment and arthroscopy

Patients with RA and PsA were recruited from the outpatient clinic at the Department of Rheumatology, St. Vincent’s University Hospital (SVUH). Arthroscopies were performed under local anaesthetic using Wolf 2.7 mm needle, and synovial tissue biopsies were obtained from the site of inflammation under direct visualisation as previously described [2, 4]. Macroscopic synovitis and vascularity were scored on a 100-mm visual analogue scale, and macroscopic vascular pattern defined as 1, 2, or 3 based on the pattern observed. A macroscopic vascular pattern demonstrating only straight regular branching vessels is defined as 1, a mixed vascular pattern displaying a mixture of straight regular branching vessels and the tortuous vessels is defined as 2, and a vascular pattern only displaying tortuous vessels is defined as 3. Clinical assessment included tender and swollen joint count (TJC/SJC), erythrocyte sedimentation rate (ESR), C-reactive protein (CRP), and global health visual analogue scale (VAS). All research was approved by the St. Vincent’s University Hospital Ethics and Medical Research Committee. Fully informed written consent was obtained from each patient prior to inclusion.

### Synovial fibroblast culture

RA, PsA, and OA biopsies were obtained at arthroscopy and digested with 1 mg/ml collagenase type I (Worthington Biochemical, Lakewood, NJ, USA) in Gibco RPMI 1640 medium (Thermo Fisher Scientific, Paisley, UK) for 4 h at 37 °C in humidified air with 5% CO_2_. Dissociated cells were plated in RPMI 1640 medium supplemented with 10% Gibco FCS (Thermo Fisher Scientific), 20 mM 4-(2-hydroxyethyl)-1-piperazineethanesulfonic acid (Thermo Fisher Scientific), penicillin (100 U/ml), streptomycin (100 U/ml), and amphotericin B (Fungizone 0.25 μg/ml; Invitrogen, Plymouth, MN, USA). SFC (1 × 10^5^) at passage 3–5 were seeded onto 6-well plates and grown to confluence. Medium was replaced, and confluent SFC were cultured for a further 24 h; this SFC conditioned medium (SFC-CM) was harvested and used for subsequent assays.

### HUVEC culture

HUVEC (Lonza, Walkerville, MD, USA) were incubated in MCDB media (Thermo Fisher Scientific) supplemented with l-glutamine (Thermo Fisher Scientific), 0.5 ml epidermal growth factor (Thermo Fisher Scientific), 50 ml FCS (Thermo Fisher Scientific), 0.5 ml of hydrocortisone, penicillin (100 U/ml; Bioscience), streptomycin (100 U/ml; Bioscience), and Fungizone (0.25 μg/ml; Bioscience). Cells were cultured at 37 °C in humidified air with 5% CO_2_ and harvested with trypsin-ethylenediaminetetraacetic acid (Lonza). Cells were used between passages 20 and 30.

### Induction of pro-angiogenic mechanisms of HUVEC in response to SFC conditioned media

To examine if RA and PsA SFC-CM can differentially affect pro-angiogenic mechanisms of HUVEC, RA and PsA SFC were cultured for 24 h at the same passage, and conditioned media (CM) was harvested. As a control culture medium, we used RPMI 1640 medium (Gibco). HUVEC were cultured with 20% RA and PsA SFC-CM for 6–24 h. Following culture, pro-angiogenic responses of endothelial cells were assessed as described in the subsections that follow.

### RNA isolation and real time quantitative PCR

Total RNA was isolated using miRNeasy Kit (Qiagen, Germany) according to the manufacturer’s specifications. To determine the gene transcript expression of pro-angiogenic mediators and putative signalling molecules in HUVEC, total RNA was reverse-transcribed and quantified using specific primers designed for VEGFA, MMP1, MMP3, MMP9, ICAM, VCAM, PFKFB3, GLUT1, and LDHA (Additional file 5: Table S1). Principal component analysis (PCA) was performed on scaled log2 expression values using the prcomp function in R (Version 3.3.1).

### HUVEC tube formation

Matrigel (50 μl; BD Biosciences, San Jose, CA, USA) was plated in 96-well culture plates after thawing on ice and allowed to polymerise for 30 min at 37 °C in humidified air with 5% CO_2_. HUVEC were removed from culture, trypsinized, and resuspended at a concentration of 3 × 10^4^ per ml in endothelial cell growth medium. Two hundred microliters of cell suspension was added to the matrigel and cultured for 8 h in the presence of 20% RA, PsA, or OA SFC-CM. HUVEC were also cultured in the presence of VEGF (25–50 ng/ml) and TSLP (50–100 ng/ml). Images were acquired from five sequential fields (× 10 magnification) as previously described [13] and using a customised version of the Angiogenesis Analyser developed for the ImageJ software (http://image.bio.methods.free.fr/ImageJ/?Angiogenesis-Analyzer-for-ImageJ).

### HUVEC wound repair assay

HUVEC were seeded onto 48-well plates and grown to confluence. A single scratch wound was induced through the middle of each well with a sterile pipette tip. Cells were subsequently stimulated for 24 h with 20% SFC-CM. HUVEC migration across the wound margins was assessed and photographed using a phase-contrast microscope. Semi-quantitative analysis of cell repopulation of the wound was assessed. Briefly, cells were fixed with 1% paraformaldehyde, stained with 0.1% crystal violet, and the number of migrating cells across the time zero margin was quantified as previously described [20].

### HUVEC surface marker expression

HUVEC were grown to confluence in 6-well plates and cultured with 20% RA SFC-CM or PsA SFC-CM for 6 h. Cells were then washed twice with cold PBS, removed by careful scraping, and incubated with human anti-VCAM-1, anti-ICAM-1, or anti-E-Selectin antibodies for 20 min at room temperature. Cells were then washed twice with PBS and fixed with 1% paraformaldehyde. Samples were acquired on a Beckman Coulter CyAn ADP flow cytometer. Flow cytometric analysis was performed using FlowJo software (FlowJo, LLC). The results were gated for mean fluorescence intensity.

### HUVEC-mediated PBMC adhesion

Peripheral blood was collected in lithium-heparin tubes (BD diagnostics), and peripheral blood mononuclear cells (PBMC) were extracted from whole blood via density gradient centrifugation using Lymphoprep (Axis-Shield, Oslo, Norway) as per the manufacturer’s instruction. Briefly, blood was diluted with an equal volume of PBS. Mixture was underlain with Lymphoprep (Nycomed, West Midlands, UK), followed by a centrifugation at 400*g* for 25 min, with brakes and acceleration turned off. The cells at the interface were collected and washed twice in sterile PBS and centrifuged at 300*g* for 5 min. HUVEC were grown to confluence in 6-well plates and treated with RPMI, 20% RA or PsA SFC-CM for 12 h. Collected PBMC were resuspended in MCDB, and 200,000 cells were applied to each well and incubated at 37 °C with 5% CO_2_ for 1 h. After the incubation time, non-adherent cells were removed by washing in sterile PBS. Semi-quantification was performed by counting the number of PBMC adherent to HUVEC in five sequential high power fields (× 10 magnification) and calculating the mean [21].

### Quantification of pro-angiogenic mediators in SFC conditioned media

To assess angiogenic vascular injury, inflammatory cytokine, and chemokine secretions from SFC-CM, a 54-plex ELISA kit spread across 7 plates was used (Meso Scale Diagnostics, USA: https://www.mesoscale.com/products/v-plex-human-biomarker-54-plex-kit-k15248d/). In this study, the multiplex kit was used to quantify in untreated PsA SFC and RA SFC supernatants the spontaneous secretions of vascular endothelial growth factor A (VEGF-A), thymic stromal lymphopoietin (TSLP), vascular endothelial growth factor receptor 1 (Flt-1), basic fibroblast growth factor (bFGF), placental growth factor (PlGF), and monocyte chemoattractant protein-1 (MCP-1). All assays were run, and SFC-CM diluted, as per the manufacturer’s recommendations for all assays except vascular injury, where a one in four dilution was performed, as per previous optimisation experiments.

### Statistical analysis

Statistical analyses were performed using Prism 5 software. For comparisons across the three experimental conditions, a one-way ANOVA was performed. In addition, the assessment of significance level for pairwise comparisons was calculated by a Mann-Whitney *U* test for analysis of non-parametric data, and Student’s two-tailed *t* test for parametric data. *p* values of less than 0.05 (*p < 0.05), 0.01 (**p < 0.01), and 0.005 (***p < 0.005) were determined as statistically significant.

## Results

### The pro-angiogenic transcriptome of PsA and RA SFC-CM-primed HUVEC are divergent

**Fig. 1:**
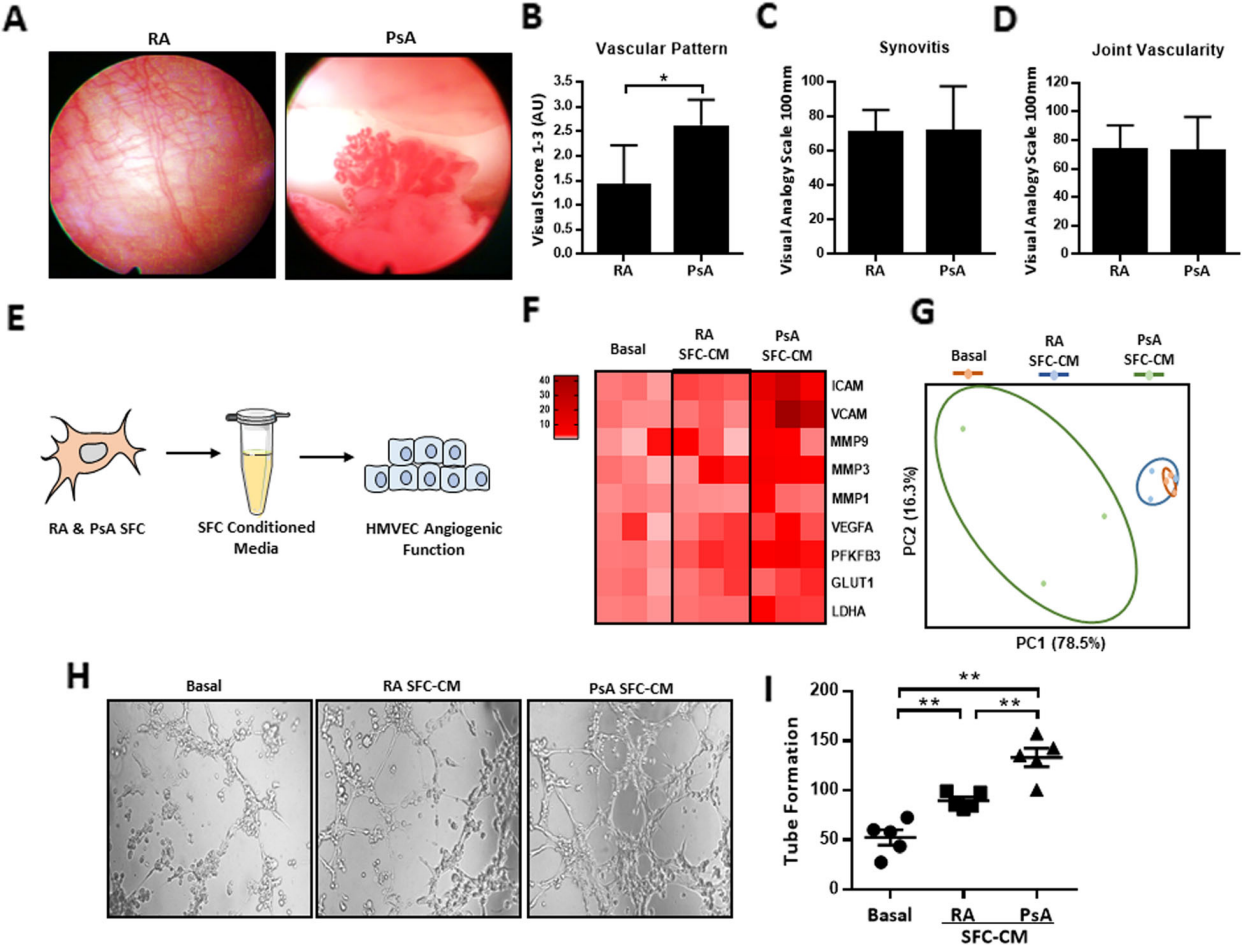
Distinct pro-angiogenic profile of PsA SFC-CM-primed HUVEC compared to RA SFC-CM. **a** Representative photomicrographs showing the distinct vascular pattern in the synovium of PsA patients as compared to RA patients. Quantification of the **b** tortuous, leaky, and bushy nature of the synovial vasculature, **c** macroscopic synovitis, and **d** angiogenesis in the synovium of RA (*n* = 7) and PsA (*n* = 8) patients. **e** Experimental setup where SFC-CM was generated from PsA and RA cultured SFCs with matched synovitis and vascular macroscopic scores but distinct vascular pattern. HUVEC cells were then cultured in the presence of control culture media, PsA SFC-CM, or RA SFC-CM and functional studies performed. **f** Heatmap displaying the gene transcripts for the key endothelial cell activation markers as quantified by RT-qPCR (basal *n* = 3, RA *n* = 5, and PsA *n* = 5); heatmap is presented as log_2_ values. **g** Principal component analysis (PCA) of gene transcripts, revealing unique clustering of PsA SFC-CM-primed HUVEC compared to both control and RA SFC-CM. **h** Representative photomicrographs showing HUVEC tube formation in response to control culture media, RA SFC-CM, and PsA SFC-CM (original magnification × 10). **i** Bar graphs quantifying the HUVEC tube formation between control culture media (*n* = 5), RA SFC-CM (*n* = 5), and PsA SFC-CM (*n* = 5). Data are expressed as mean ± SEM. *p < 0.05, **p < 0.01, and *** *p* < 0.005 were considered significantly different

Previous studies have demonstrated distinct synovial vascular morphology in PsA compared to RA; however, the underlying mechanisms involved are still unclear [2, 4]. In this study, we further investigated whether the RA and PsA joint microenvironment contributes to the observed distinct vascular patterns. Consistent with previous reports, the synovium of patients with PsA displayed a distinct vascular morphology, with higher observations of tortuous elongated and dilated vessels compared to the synovium of patients with RA (Fig. 1a, b). To ensure that any differences in angiogenic function were independent of differences in disease severity, SFC were isolated from RA and PsA patients with matched scores of macroscopic synovitis (Fig. 1c) and vascularity (Fig. 1d). Using SFC-CM derived from these matched RA and PsA patients, we examined how healthy endothelial cells respond to RA SFC-CM and PsA SFC-CM (Fig. 1e). Transcriptome analysis showed induction of pro-angiogenic regulators in PsA SFC-CM-primed HUVEC compared to both RA SFC-CM and control culture media (Fig. 1f). Principal component analysis (PCA) revealed a distinct molecular response between endothelial cells exposed to PsA SFC and RA SFC microenvironments, respectively (Fig. 1g).

### SFC-CM promotes HUVEC tube formation

To assess the effect of RA and PsA SFC-CM on angiogenic tube formation, matrigel tube formation assays were performed. HUVEC were cultured with control culture media, RA SFC-CM, or PsA SFC-CM for 6 h, and HUVEC tube formation was quantified using phase-contrast microscopy. Figure 1h demonstrates the effect of control culture media, RA, or PsA SFC-CM on HUVEC formation of tube-like structures. Figure 1i graphically illustrates markedly induced tube formation in response to RA (*p* < 0.05) and PsA SFC-CM (*p* < 0.01) treatment as compared to control culture media, with PsA SFC-CM showing a greater effect than RA SFC-CM (*p* < 0.05). Finally, OA SFC-CM had minimal effect on EC tube-like structures compared to that of PsA SFC-CM (Additional file 1: Figure S1).

### SFC-CM promotes HUVEC migration

**Fig. 2:**
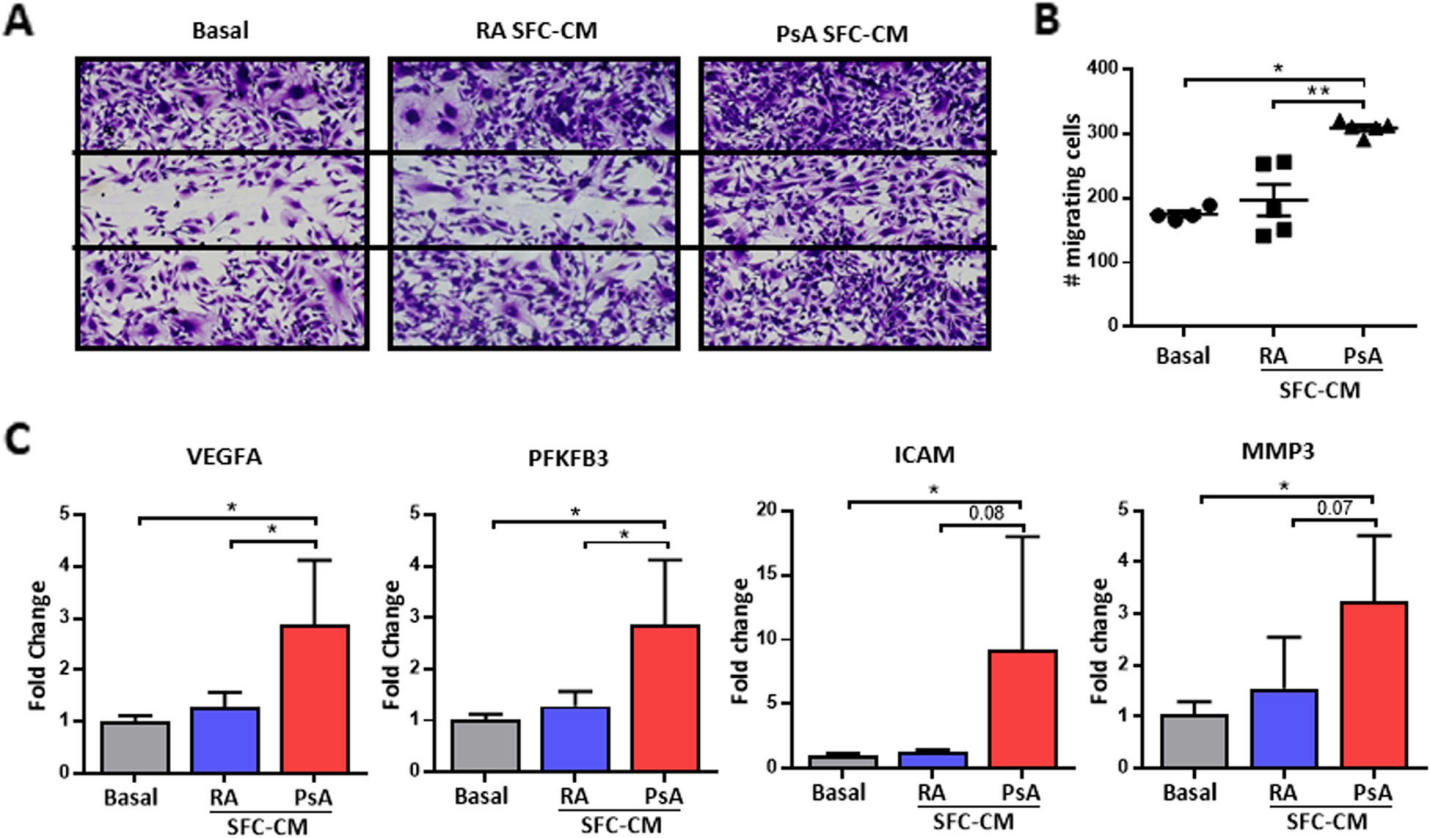
PsA SFC-CM-induced HUVEC migration. **a** Representative photomicrographs show HUVEC migration following culture with control culture media, RA SFC-CM, and PsA SFC-CM (magnification × 10). **b** Representative graph quantifying HUVEC migration following stimulation with control culture media (*n* = 3), RA SFC-CM (*n* = 5), and PsA SFC-CM (*n* = 5). **c** RT-qPCR analysis of VEGFA, PFKFB3, ICAM, and MMP3 expression in control (*n* = 3), PsA SFC-CM- (*n* = 5), and RA SFC-CM-primed (*n* = 5) HUVEC. Data are expressed as mean ± SEM. *p < 0.05, **p < 0.01 significantly different

To examine the functional significance of RA and PsA SFC-CM on HUVEC migration, HUVEC were cultured in the presence of control culture media, RA SFC-CM, or PsA SFC-CM following wound induction. Figure 2a demonstrates the markedly induced cell migration across the wound area in HUVEC treated with PsA SFC-CM compared to control culture media. No significant increase in HUVEC migration was observed in response to RA SFC-CM compared to control culture media. PsA SFC-CM significantly induced HUVEC migration compared to RA SFC-CM (*p* < 0.01) (Fig. 2b). This was paralleled by a significant increase in mRNA expression levels of VEGFA, PFKFB3, ICAM, and MMP3 in PsA SFC-CM-treated HUVEC compared to control (all *p* < 0.05). Expression of VEGFA and PFKFB3 mRNA were significantly higher in PsA SFC-CM compared to RA SFC-CM treatment (*p* < 0.05) (Fig. 2c).

### PsA SFC-CM significantly increases HUVEC adhesion molecule expression

**Fig. 3:**
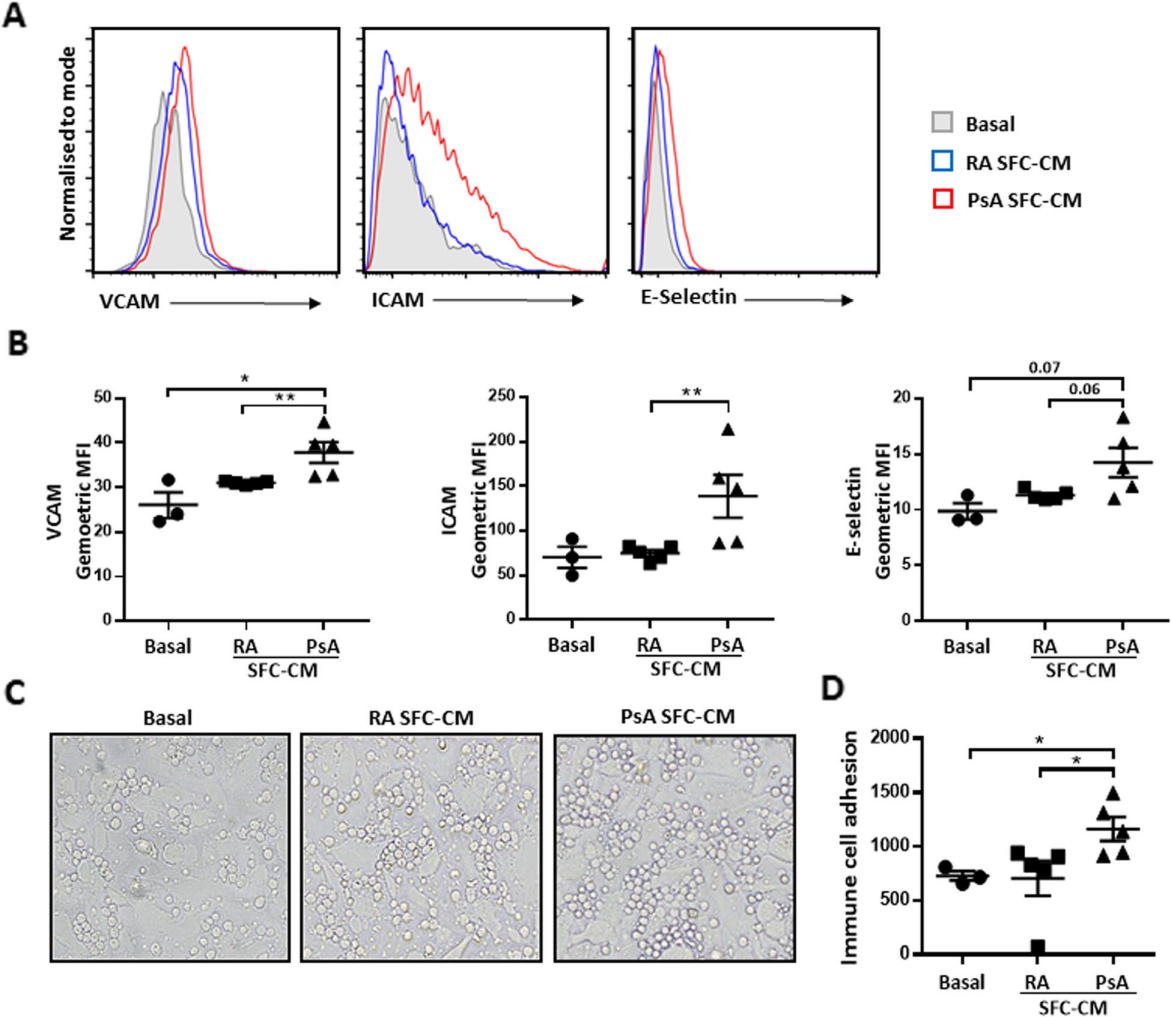
SFC-CM-induced surface expression of adhesion markers and immune cell adhesion to HUVEC. **a** Representative histograms of surface adhesion marker expression (VCAM, ICAM, and E-Selectin) on HUVEC in response to control culture media, RA SFC-CM, and PsA SFC-CM. **b** Bar graphs quantifying the surface expression of VCAM, ICAM, and E-Selectin following exposure to control culture media (*n* = 3), RA SFC-CM (*n* = 5), and PsA SFC-CM (*n* = 5). **c** Representative photomicrographs of PBMC adhesion to HUVEC in response to control culture media (*n* = 3), RA SFC-CM (*n* = 5), and PsA SFC-CM (*n* = 5). **d** Bar graph representing corresponding semi-quantification of PBMC adhesion to HUVEC. Data are expressed as mean ± SEM. *p < 0.05, **p < 0.01 significantly different

Dysfunctional angiogenesis is known to promote synovial inflammation by allowing for immune cell infiltration from the circulation to the site of inflammation. With this is mind, we next examined if HUVEC activation by SFC-CM can also increase adhesion molecule expression and therefore influence immune cell adhesion. Flow cytometric analysis was performed to assess the effect of RA and PsA SFC-CM on HUVEC expression of adhesion molecules, VCAM-1, ICAM-1, and E-Selectin, following a 6-h incubation period (Fig. 3a). Quantification bar graphs in Fig. 3b demonstrated increased surface expression of VCAM-1 (*p* < 0.05) and ICAM-1 (*p* < 0.05) in PsA SFC-CM-treated cells compared to control culture media. PsA SFC-CM also significantly increased VCAM-1 (*p* < 0.01) and ICAM-1 (*p* < 0.01) expression compared to RA SFC-CM. E-Selectin expression was also increased in PsA SFC-CM-treated HUVEC, compared to both control and RA SFC-CM treatment; however, this did not reach statistical significance (*p* = 0.0714 and *p* = 0.0635, respectively). No significant effect was observed for all three adhesion molecules in response to RA SFC-CM compared to control culture media.

### PsA SFC-CM significantly increases PBMC adhesion to HUVEC

Because we found a significant increase in adhesion molecule expression on the surface of HUVEC in response to PsA SFC-CM, we next examined the effect of PsA SFC-CM on immune cell adhesion to HUVEC. Healthy control PBMC were incubated for 1 h on confluent HUVEC monolayer cultures pre-treated for 12 h with control culture media, RA SFC-CM, or PsA SFC-CM. Adhesion was assessed by counting the number of adherent PBMC to the HUVEC monolayer. Representative photomicrographs in Fig. 3c demonstrate the effect of control culture media, RA, and PsA SFC-CM on PBMC adhesion. Semi-quantitative analysis in Fig. 3d shows no significant increase in PBMC adhesion to HUVEC monolayers in response to RA SFC-CM compared to control culture media-treated cells. In contrast to this, PsA SFC-CM significantly increased PBMC adhesion compared to control culture media (*p* < 0.01) and RA SFC-CM (*p* < 0.01).

### Quantification of soluble pro-angiogenic mediators in PsA SFC-CM and RA SFC-CM

**Fig. 4:**
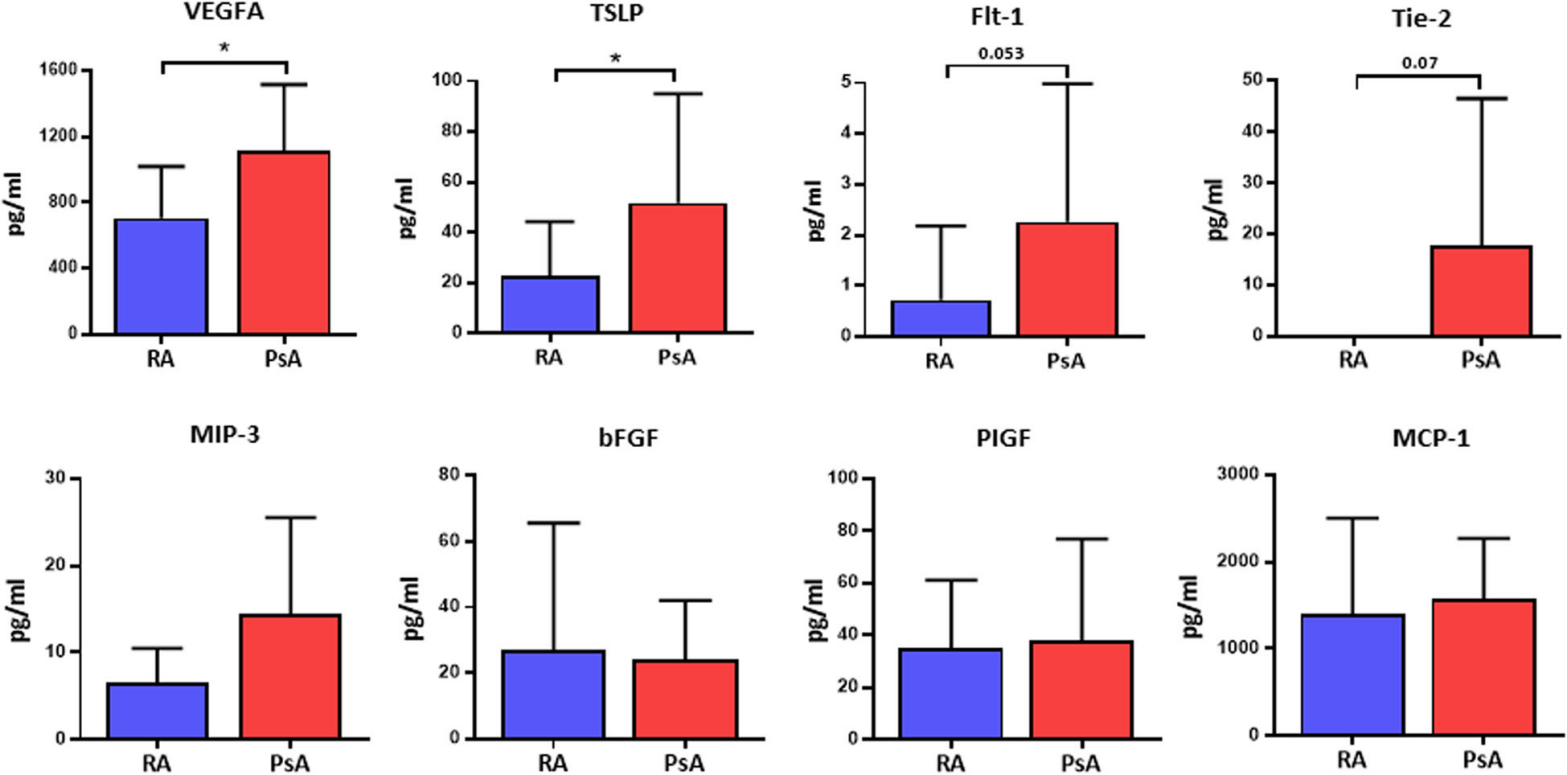
Expression of pro-angiogenic mediators in PsA and RA SFC-CM. Quantification of VEGFA, TSLP, Flt-1, Tie-2, MIP-3, bFGF, PIGF, and MCP-1 in the CM derived from RA (*n* = 10) and PsA (*n* = 10) SFC. Data are expressed as mean ± SEM. *p < 0.05 significantly different

We next examined the concentration of soluble pro-angiogenic mediators in RA SFC-CM and PsA SFC-CM to determine if there was any difference in factors that promote endothelial cell activation. Figure 4 demonstrates increased secretion of key angiogenic mediators, VEGFA, TSLP, Flt-1, and Tie-2, from untreated PsA SFC compared to RA SFC, yet other angiogenic mediators remain unchanged between the PsA SFC and RA SFC (Fig. 4; Additional file 2: Figure S2). These data support a concept of complex interplay between SFC secretion of pro-angiogenic mediators and synovial angiogenesis, which may contribute to the unique vascular pattern observed in PsA. Finally, we demonstrated that VEGF and TSLP directly induce endothelial cell tube formation (Additional file 3: Figure S3).

## Discussion

In this study, we demonstrate, for the first time, that soluble factors from PsA and RA synovia differentially regulate angiogenesis and endothelial cell function which may contribute to the distinct synovial vascular patterns observed in the PsA and RA synovium. The observed changes were independent of differences in synovial inflammation and overall joint vascularity as the SFC-CM were generated from patients with matched synovial inflammation and vascularity, but different vascular pattern. A molecular comparison of SFC-CM-primed HUVEC revealed a disease-specific pro-angiogenic transcriptome with elevated expression of MMPs, adhesion molecules, and pro-angiogenic mediators in response to PsA SFC-CM compared to RA SFC-CM. These molecular differences functionally influenced HUVEC angiogenic mechanisms, as reflected by increased HUVEC tube formation and migration in response to PsA SFC-CM compared to RA SFC-CM. PsA SFC-CM also induced the surface expression of adhesion molecules, VCAM, ICAM, and E-Selectin on HUVEC compared to RA SFC-CM. This was paralleled by changes in cell adhesion, with PsA SFC-CM inducing greater immune cell adhesion to HUVEC as compared to RA SFC-CM. Finally, we identified that CM from PsA SFC displayed higher levels of VEGFA, TSLP, Flt-1, and Tie2 compared to RA SFC-CM, suggesting that the PsA joint microenvironment contributes to the distinct vascular morphology observed in the inflamed joint.

Synovial fibroblasts are central players in synovial inflammation and are associated with the secretion of pro-inflammatory mediators, proteases, and pro-angiogenic factors [12, 22]. We and others have previously shown that fibroblasts, in response to cytokines, oxidative stress, and TLR ligands, induce the secretion of pro-angiogenic mediators including VEGF, EGF, Tie2, Ang1, and Ang2 [13, 14, 23–26]. The dynamic interactions between the SFC-derived pro-angiogenic mediators and endothelial cells are critical for new vessel formation, stability, and survival. In the present study, using SFC-CM from RA and PsA patients with matched macroscopic joint vascularity, in the absence of additional stimuli, PsA SFC-CM induced a molecularly distinct pro-angiogenic phenotype in HUVEC as compared to control culture media or RA SFC-CM. In particular, PsA SFC-CM-primed HUVEC displayed increased expression of VEGFA, PFKFB3, MMP1, MMP3, and MMP9, suggesting that the SFC-induced microenvironment differentially modulates endothelial cell activity. This effect is independent of disease severity and may contribute, at least in part, to the distinct vascular morphology observed in the PsA synovium. Consistent with this hypothesis is the observation of increased expression of VEGFA, cytokines, and MMPs in the synovium of early PsA patients compared to RA patients and the associated vascular morphology [7–9, 27] in addition to other growth factors such as Ang2, PIGF, and SDF-1 [10, 22]. In turn, VEGF, Ang2 [28], and hypoxia [29] are known to induce the DLL-4-Notch signalling pathway, which is pivotally involved in promoting angiogenesis [28].

More recently, PFKFB3-driven glycolysis has been associated with pathologic angiogenesis. Under conditions of hypoxia and inflammation, the PFKFB3-encoded enzyme, PFK-2/FBPase, activates a key irreversible enzyme, 6-phosphofructo-1-kinase (PFK-1), resulting in untethered glycolysis [30, 31], which reportedly results in a hyper-branching vascular phenotype [32]. Consistent with this, the metabolic profile of tumour-derived endothelial cells, which are phenotypically very similar to those in the PsA synovium, revealed hyper-glycolytic activity compared to endothelial cells derived from healthy tissue [33], an effect largely attributed to the tumour-associated microenvironment.

The molecular analysis supports our findings of functional alterations including increased tube formation, migration, and expression of cell surface markers in PsA SFC-CM-primed HUVEC compared with those primed with control culture media or RA SFC-CM. In parallel, we demonstrated increased immune cell adhesion in PsA SFC-CM-primed HUVEC compared to control culture media and RA SFC-CM, confirming a distinct shift in endothelial cell activation. In support of this, expression of pro-angiogenic mediators such as VEGF correlate with macroscopic vascularity in PsA [7]. Furthermore, previous studies have demonstrated vascular regression, decreased growth factor expression, and reduced immune cell infiltration in the skin and synovium of psoriasis and PsA patients, respectively, in parallel with clinical improvement following anti-TNF-α therapy [10, 34–38]. Interestingly, RA SFC-CM-primed HUVEC displayed enhanced tube formation compared to basal control but had no significant effect on HUVEC migration, cell surface adhesion marker expression, or immune cell adhesion (Additional file 4: Figure S4). Previous studies have demonstrated that RA SFC-derived CM induces endothelial cell activation [13, 39]; however, these observations were demonstrated following exposure of the RA SFC to pro-inflammatory stimuli or hypoxia, before co-culture with endothelial cells. These differences suggest that the PsA SFC are intrinsically more primed for pro-angiogenic activity, even in the absence of exogenous pro-inflammatory stimuli.

Multiple pro-angiogenic factors have been found to be overexpressed by inflammatory fibroblasts, which could contribute to dysfunctional angiogenesis at the site of inflammation [12, 39–41]. Increased constitutive expression of VEGF observed in untreated PsA SFC-CM compared to RA SFC-CM partly explains the observed differences in pro-angiogenic activity of the SFC-CM-treated endothelial cells. Consistent with this, VEGF and Ang2, which synergistically induce the Notch–Dll4 interaction to control vessel sprouting, are more highly expressed in the synovium in PsA compared with that in RA and OA [28]. The crucial role of fibroblast-derived VEGF in vessel sprouting is further emphasised by the inability of VEGF-deficient mouse embryonic fibroblasts to promote tumour vascularisation in immune-deficient mice [41]. However, increased constitutive VEGF expression has not been described previously in cultured PsA SFC compared to RA SFC. While this study demonstrates the effect of PsA and RA SFC-CM on endothelial cell function, a limitation of the study is that primary normal synovial microvascular endothelial cells were not utilised, which would be more reflective of the joint. This is due to the difficulty of isolating and culturing synovial endothelial cells from normal synovium. Previous studies have used non-synovial primary microvascular cells as a model; however, using a different vascular bed does not reflect the joint microenvironment. Indeed, recent studies examining other stromal cells in the joint such as fibroblasts show that even within the synovium, there are many distinct subsets within one joint with distinct functions [42]; in addition, this can also vary depending on whether the cells are obtained from large or small joints [43]. Recent advances in cell sorting may lead to better methodologies for isolating primary synovial EC and thus lead to models that more closely reflect the joint microenvironment.

Interestingly, TSLP was also significantly elevated in PsA SFC-CM, suggesting that in addition to elevated VEGF and the associated receptors, TLSP is implicated in the distinct PsA SFC-induced vascular phenotype. Previous studies show that tumour-derived TSLP interacts with endothelial cells to promote angiogenesis in cervical cancer [44, 45], resulting in the activation of the downstream PI3K/AKT pathway [46]. Recently, TSLP has been implicated as a disease-promoting factor in RA, with elevated levels being observed in the synovial fluid of patients with RA as compared to OA [47]. In a model of collagen-induced arthritis, administration of TSLP significantly exacerbated disease severity scores and joint damage with increased T cell infiltration [48]. Interestingly, increased TSLP expression has not been described previously in PsA. It is also noteworthy that other pro-angiogenic factors such as bFGF, P1GF, and MCP-1 were indistinguishable across the disease groups, suggesting the pro-angiogenic effects of PsA and RA SFC are heterogeneous and act through different molecular pathways. However, while both PsA and RA fibroblasts have been previously described to enhance pro-angiogenic activity, this is the first time a differential, disease-specific process has been demonstrated. Whether the effect of these molecules on endothelial cell function is due to their secretion from SFC as soluble mediators or through vesicular transfer is unclear. Previous studies have demonstrated increases in extracellular vesicles (EV), including exosomes and microparticles, in the inflamed joint which can regulate synovial fibroblasts and osteoclast function [49, 50]. Studies have also shown that synoviocyte fibroblasts secrete EV which contain TNF-α, VEGF, IL-6, and MMP3 suggesting that EV secretion may be one of the mechanisms by which these molecules induce angiogenic responses [51, 52].

While we have demonstrated VEGF and TLSP are increased in PsA SFC-CM compared to RA SFC-CM, the upstream mechanisms that drive the differential secretion of angiogenic factors from PsA vs RA SFC are not clear. Both PsA and RA differ in their responses to specific anti-cytokine therapies, such as anti-IL-17A, anti-IL-12/23, and anti-IL-6; thus, differential cytokine regulation of angiogenic growth factors may be involved. Another possible mechanism may relate to hypoxia, which has been previously reported to be more severe in PsA compared to RA [53]. Finally, it may reflect genetic or epigenetic effects, as differences have also been described between PsA and RA in terms of these factors. Studies to date have shown differences in the epigenetic regulation of RA SFC invasive mechanisms depending on the joint site [42], in addition to differential transcriptional regulation in specific RA SFC subpopulations [43]; however, similar studies have yet to be performed in PsA SFC.

## Conclusion

This is the first study to show that SFC-mediated pro-angiogenic mechanisms are greater in PsA compared to RA, thus supporting the targeted inhibition of SFC-derived pro-angiogenic factors as an alternative approach to reducing the impact of dysfunctional angiogenesis on inflammatory arthritis.

## Supplementary information

**Additional file 1 : Figure S1.** Representative photomicrographs showing HUVEC tube formation in response to control culture media, RA SFC-CM, PsA SFC-CM and OA SFC-CM (original magnification × 10). Dot plots quantifying the HUVEC tube formation between control culture media (*n* = 2), RA SFC-CM (*n* = 2), PsA SFC-CM (*n* = 2), and OA SFC-CM (*n* = 2). Data are expressed as mean ± SEM.

**Additional file 2 : Figure S2.** Quantification of pro-inflammatory mediators in the untreated CM derived from RA (*n* = 10) and PsA (*n* = 10) SFC. Data are expressed as mean ± SEM. *p < 0.05 significantly different.

**Additional file 3: Figure S3.** Representative photomicrographs showing HUVEC tube formation in response to VEGF (25 ng/ml and 50 ng/ml) and TLSP (50 ng/ml and 100 ng/ml) (original magnification × 10). (A) Dot plots quantifying the HUVEC tube formation in response to VEGF and TLSP (*n* = 3). Data are expressed as mean ± SEM.

**Additional file 4 : Figure S4.** (A) Photomicrographs showing individual HUVEC migration following culture with control culture media (*n* = 3), RA SFC-CM (*n* = 3) and PsA SFC-CM (*n* = 3).

**Additional file 5 : Table S1.** RT-qPCR primer sequences.

## Data Availability

All data generated or analysed during this study are included in this published article (and its Additional files).

## Abbreviations

ANG2: Angiopoietin-2
bFGF: Basic fibroblast growth factor
CM: Conditioned media
CRP: C-reactive protein
ESR: Erythrocyte sedimentation rate
Flt-1: Vascular endothelial growth factor receptor 1
HUVEC: Human umbilical vein endothelial cells
IA: Inflammatory arthritis
MCP-1: Monocyte chemoattractant orotein-1
MMPs: Matrix metalloproteinases
PBMC: Peripheral blood mononuclear cells
PIGF: Placental growth factor
PsA: Psoriatic arthritis
RA: Rheumatoid arthritis
SFC: Primary synovial fibroblast cells
SJC: Swollen joint count
SVUH: St. Vincent’s University Hospital
TJC: Tender joint count
TSLP: Thymic stromal lymphopoietin
VAS: Visual analogue scale
VEGF: Vascular endothelial growth factor
