# Supplementary figures and images for: Enhanced angiogenic function in response to fibroblasts from psoriatic arthritis synovium compared to rheumatoid arthritis
Source: Arthritis Res Ther. 2019 Dec 21;21:297. doi: 10.1186/s13075-019-2088-3 (PMC6925847; doi:10.1186/s13075-019-2088-3)

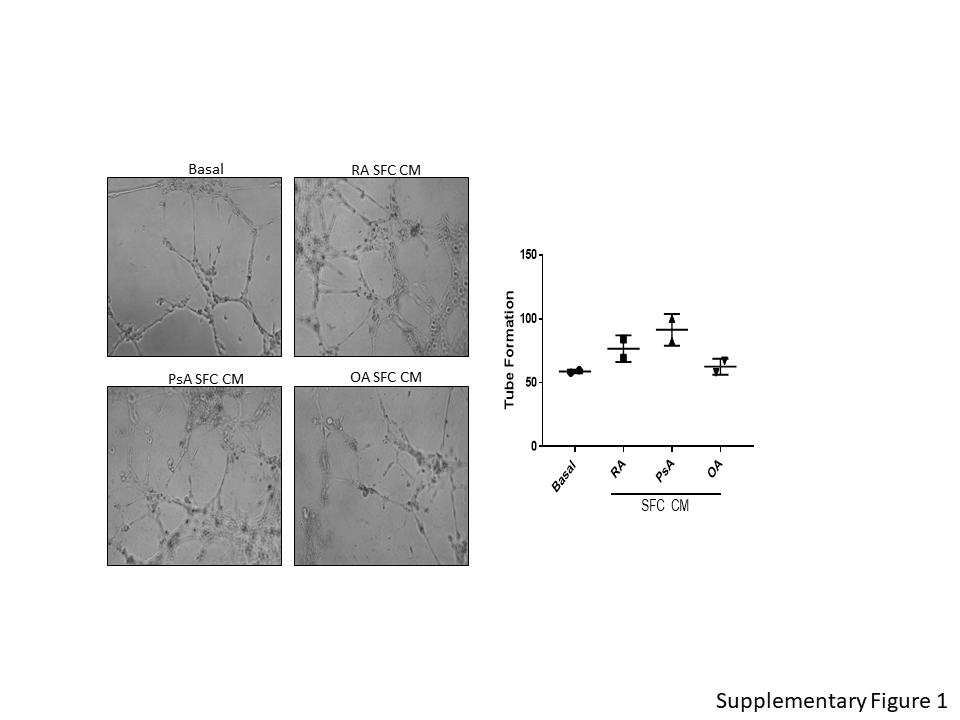

Supplement: Supplementary file 1 — Additional file 1 : Figure S1. Representative photomicrographs showing HUVEC tube formation in response to control culture media, RA SFC-CM, PsA SFC-CM and OA SFC-CM (original magnification × 10). Dot plots quantifying the HUVEC tube formation between control culture media (n = 2), RA SFC-CM (n = 2), PsA SFC-CM (n = 2), and OA SFC-CM (n = 2). Data are expressed as mean ± SEM. [file 13075_2019_2088_MOESM1_ESM.tif]

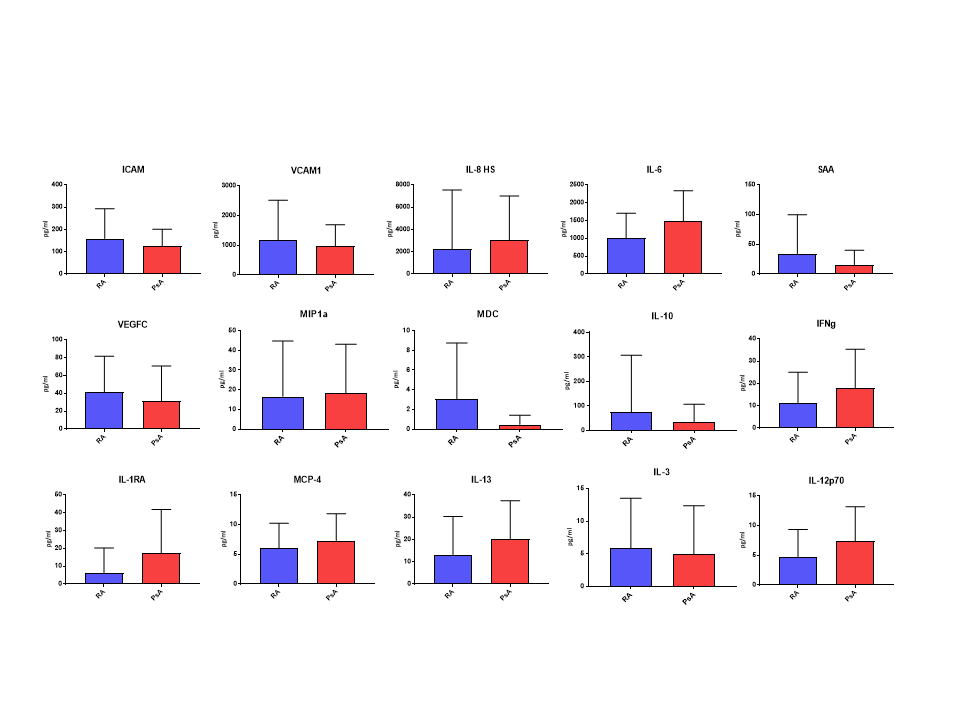

Supplement: Supplementary file 2 — Additional file 2 : Figure S2. Quantification of pro-inflammatory mediators in the untreated CM derived from RA (n = 10) and PsA (n = 10) SFC. Data are expressed as mean ± SEM. *p < 0.05 significantly different. [file 13075_2019_2088_MOESM2_ESM.tif]

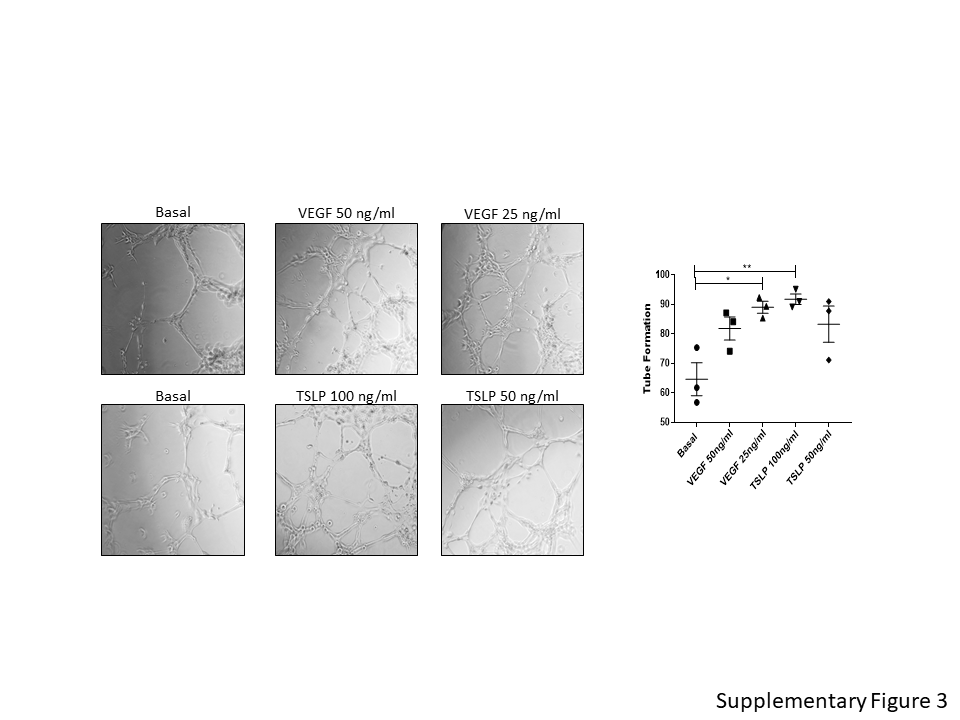

Supplement: Supplementary file 3 — Additional file 3: Figure S3. Representative photomicrographs showing HUVEC tube formation in response to VEGF (25 ng/ml and 50 ng/ml) and TLSP (50 ng/ml and 100 ng/ml) (original magnification × 10). (A) Dot plots quantifying the HUVEC tube formation in response to VEGF and TLSP (n = 3). Data are expressed as mean ± SEM. [file 13075_2019_2088_MOESM3_ESM.tif]

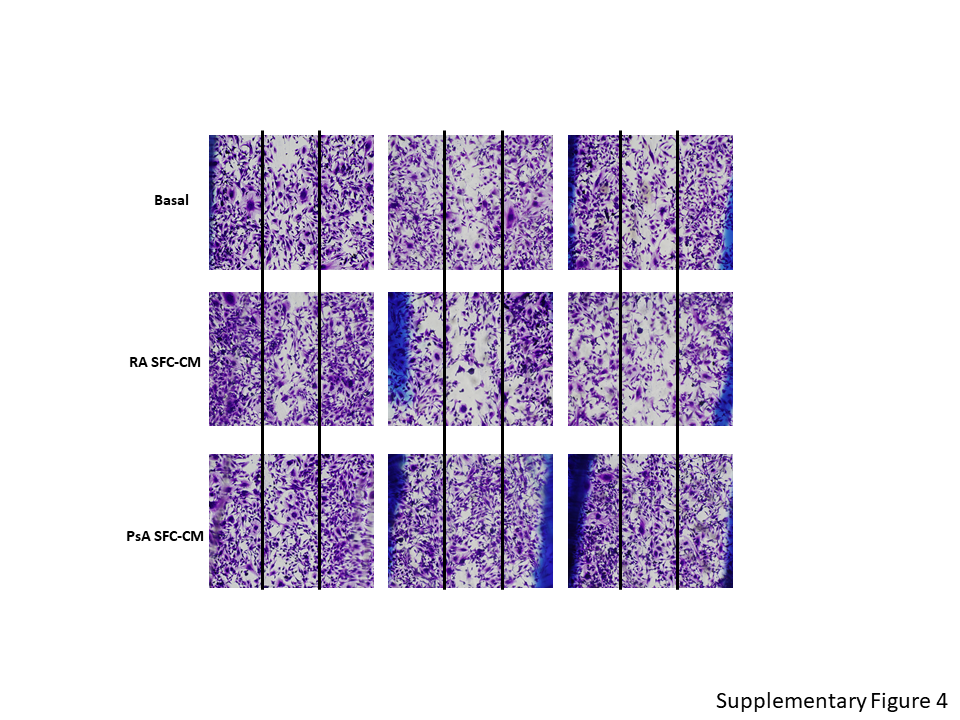

Supplement: Supplementary file 4 — Additional file 4 : Figure S4. (A) Photomicrographs showing individual HUVEC migration following culture with control culture media (n = 3), RA SFC-CM (n = 3) and PsA SFC-CM (n = 3). [file 13075_2019_2088_MOESM4_ESM.tif]
