# Supplementary material for: Enhanced angiogenic function in response to fibroblasts from psoriatic arthritis synovium compared to rheumatoid arthritis
Source: Arthritis Res Ther. 2019 Dec 21;21:297. doi: 10.1186/s13075-019-2088-3 (PMC6925847; doi:10.1186/s13075-019-2088-3)
Supplement: Supplementary file 5 — Additional file 5 : Table S1. RT-qPCR primer sequences. [file 13075_2019_2088_MOESM5_ESM.docx]

Table S1: RT-qPCR primer sequences
